# Anæsthesia from Chloroform Prolonged by the Hypodermic Injection of Morphia

**Published:** 1864-05

**Authors:** 


### ANÆSTHESIA FROM CHLOROFORM PROLONGED BY THE HYPODERMIC INJECTION OF MORPHIA.

TRANSLATED BY DR. HOMBURG, CINCINNATI.

The following observations of Prof. Nupbaum, of Munich, are likely to prove of vast importance not only in chirurgical, but also for internal medical treatment, for instance, in reference to the therapy of the tetanus, various neuroses, etc., yea, even in experimental physiology. Since it appears to us desirable that the valuable experiments in question should be confirmed by other surgeons and physicians so that experiments may be had in the most varied manner, we hasten to communicate them briefly, even without waiting for a greater number of cases bearing thereon.

Prof. Nupbaum removed, about three weeks ago, from a patient aged forty, a miller, residing in Foelz, a great sarcomatous tumor on the neck, using chloroform in the usual manner. To silence pains after the operation, which required a complete separation of plexus cervicalis, he injected beneath his skin, while still under the influence of chloroform, one grain of acetate of morphine. The person operated upon did not subsequently—as usual—awaken from his narcotism, but slept on, breathing regularly and calmly, uninterruptedly, for twelve hours. He endured during this sleep the deepest stitches of the needle, incisions into the skin, and the application of red-hot iron, etc., without even the slightest re-action against the same. Finally, he awoke ftom deep slumber, exactly as if he had just passed through a chloroform narcotism.

A few days later, Prof. Nupbaum most pleasingly surprised at this exhibition, and the effect just stated of subcutaneous application of morphine on a second patient, a Mr. M., in Swabia, upon whom, in consequence of a cancer, he had just executed the resection of the upper maxillary bone without removing the alveolar process during the chloroform narcotism, and had finally, on account of cancerous irritation in the facial skin, undertaken a transplantation in the neighborhood of the temples and forehead by closing the wound. This patient, too, slept with complete absence of all feeling during eight hours amid the most quiet breathing. His pulse remained in rhythm and number perfectly regular. The effect of the narcotic appears the more surprising in this case, because the same dose of acetate of morphine had a few days previous been injected hypodermically without producing sleep, and still less anæsthesia.

Two other cases embrace a woman fifty years old, and a seven year old boy, upon both of whom only about half a grain of morphine had been subcutaneously injected; and both slept from five to six hours the same quiet sleep, and enjoyed an equal anæsthetic condition. Another case, in which the experiment in question failed, has up to now not been observed by Professor Nupbaum.

From the preceding observations appears to arise a physiological experimental point, that must on further use tend doubtless to most gratifying results. Obviously it appears as if the hypodermic application of morphine, and perhape of other narcotics, for instance, of atrophia, might during the chloroform narcose preserve for several (six to twelve) hours that peculiar condition of the central nervous system, of which we know—it is to be lamented—as yet so little, and which is temporarily produced by the effect of inhaled chloroform, and to do this by greater or lesser doses of morphine; as long at least as the effect of morphine is maintained; and of course also the arracothesy, which to produce through the inhalation of chloroform is, as well known, one of the most beneficent inventions in aid of suffering humanity.—*Cincinnati Lancet and Observer.*

